# Expression profiles and potential functions of long noncoding RNAs and mRNAs in autoimmune pulmonary alveolar proteinosis patients

**DOI:** 10.18632/aging.202818

**Published:** 2021-04-04

**Authors:** Yanli Yang, Wenshuai Xu, Ruo-Lan Xiang, Xinlun Tian, Kai-Feng Xu

**Affiliations:** 1 Department of Pulmonary and Critical Care Medicine, Peking Union Medical College Hospital, Chinese Academy of Medical Sciences & Peking Union Medical College, Beijing 100730, China; 2 Department of Physiology and Pathophysiology, Peking University School of Basic Medical Sciences, Beijing 100191, China

**Keywords:** autoimmune pulmonary alveolar proteinosis, long noncoding RNAs, microarray, network

## Abstract

Autoimmune pulmonary alveolar proteinosis (APAP) is a rare lung disease that may be associated with surfactant overaccumulation. To assess the function of long noncoding RNAs (lncRNAs) in APAP, we performed microarray analyses to identify differentially expressed (DE) lncRNAs and mRNAs between peripheral blood samples from five APAP patients and five healthy volunteers. In total, 12459 DE lncRNAs and 9331 DE mRNAs were identified in APAP patient samples. A qRT-PCR validation of 20 DE lncRNAs and 20 mRNAs indicated that 12 DE lncRNAs may be involved in the pathogenesis of APAP. Coding and noncoding co-expression (CNC) and competing endogenous RNA (ceRNA) regulatory networks were constructed with these 12 DE lncRNAs. Gene Ontology analysis of the downregulated mRNAs and the CNC network revealed that “ubiquitin-like protein transferase activity” was suppressed in APAP patient samples. Kyoto Encyclopedia of Genes and Genomes analysis demonstrated that the “MAPK signaling pathway” was enriched in the ceRNA network. Gene Ontology analysis also indicated that mRNAs involved in many transmembrane ion transport processes were upregulated in APAP patients. The DE lncRNAs and mRNAs discovered in this study have elucidated the pathogenesis of APAP, and the CNC and ceRNA networks have provided novel insights for future functional research.

## INTRODUCTION

Pulmonary alveolar proteinosis is a rare diffuse lung disease characterized by overaccumulation of surfactants in the alveoli and bronchiole terminals [1]. The annual prevalence of this disease is estimated to be 3.7–6.2 cases per million people [2, 3]. Autoimmune pulmonary alveolar proteinosis (APAP) is the main variant of pulmonary alveolar proteinosis, accounting for 91% of cases [4].

APAP is characterized by the production of autoantibodies against granulocyte-macrophage colony-stimulating factor (GM-CSF). These autoantibodies have been detected at high levels in serum and bronchoalveolar lavage fluid samples from APAP patients. GM-CSF is responsible for surfactant catabolism and homeostasis in alveolar macrophages, whereas GM-CSF autoantibodies prevent the clearance of pulmonary surfactants by alveolar macrophages and induce the accumulation of lipoprotein-rich materials [5, 6]. This accumulation restricts pulmonary ventilation, reduces the lung diffusion capacity and can even lead to respiratory failure.

Whole-lung lavage is the standard treatment for APAP and can improve lung function in most patients. The whole-lung lavage procedure is performed under general anesthesia using a double-lumen endotracheal tube to ventilate one lung while repeatedly filling and emptying the other lung with up to 50 L of saline to physically remove surfactants from it. However, repeated treatments are usually required due to the re-accumulation of surfactants. Moreover, the use of whole-lung lavage therapy is limited because it is an invasive procedure that requires anesthesia [7]. Therefore, more effective treatments need to be explored. However, the pathogenesis of APAP is not completely understood, presenting an obstacle to the development of new treatments.

Long noncoding RNAs (lncRNAs) are a class of RNAs that are > 200 nucleotides long and do not encode proteins. By binding to DNA, RNA or proteins, lncRNAs regulate the expression of genes involved in various cellular processes, genome regulatory networks and diseases [8]. LncRNAs are associated with autoimmune diseases such as systemic lupus erythematosus, rheumatoid arthritis, Sjögren’s syndrome, etc. For instance, two lncRNAs (linc0949 and linc0597) were found to be significantly downregulated in peripheral blood mononuclear cells from patients with systemic lupus erythematosus, and linc0949 was proposed to be a biomarker of the disease [9]. Noncoding transcript in T cells, a lncRNA in monocytes, was reported to be hyperactivated in peripheral blood mononuclear cells from early rheumatoid arthritis patients, whereas its expression was found to decline significantly after treatment [10]. The lncRNA PICSAR was found to promote synovial invasion and joint destruction by sponging miR-4701-5p in fibroblast-like synoviocytes from rheumatoid arthritis patients, and is likely to be used as a biomarker of rheumatoid arthritis [11]. Although lncRNAs are involved in several autoimmune diseases, it is unclear whether they are dysregulated in APAP.

To investigate the involvement of lncRNAs in APAP, we compared the expression profiles of lncRNAs and mRNAs in peripheral blood samples from five APAP patients and five matched healthy controls. We then used bioinformatics to analyze lncRNA-mRNA and lncRNA-microRNA (miRNA)-mRNA regulatory networks. Our identification of differentially expressed (DE) lncRNAs and their potential corresponding mRNAs has provided insight into the pathogenesis of APAP.

## RESULTS

### Expression profiles of lncRNAs and mRNAs

**Figure 1:**
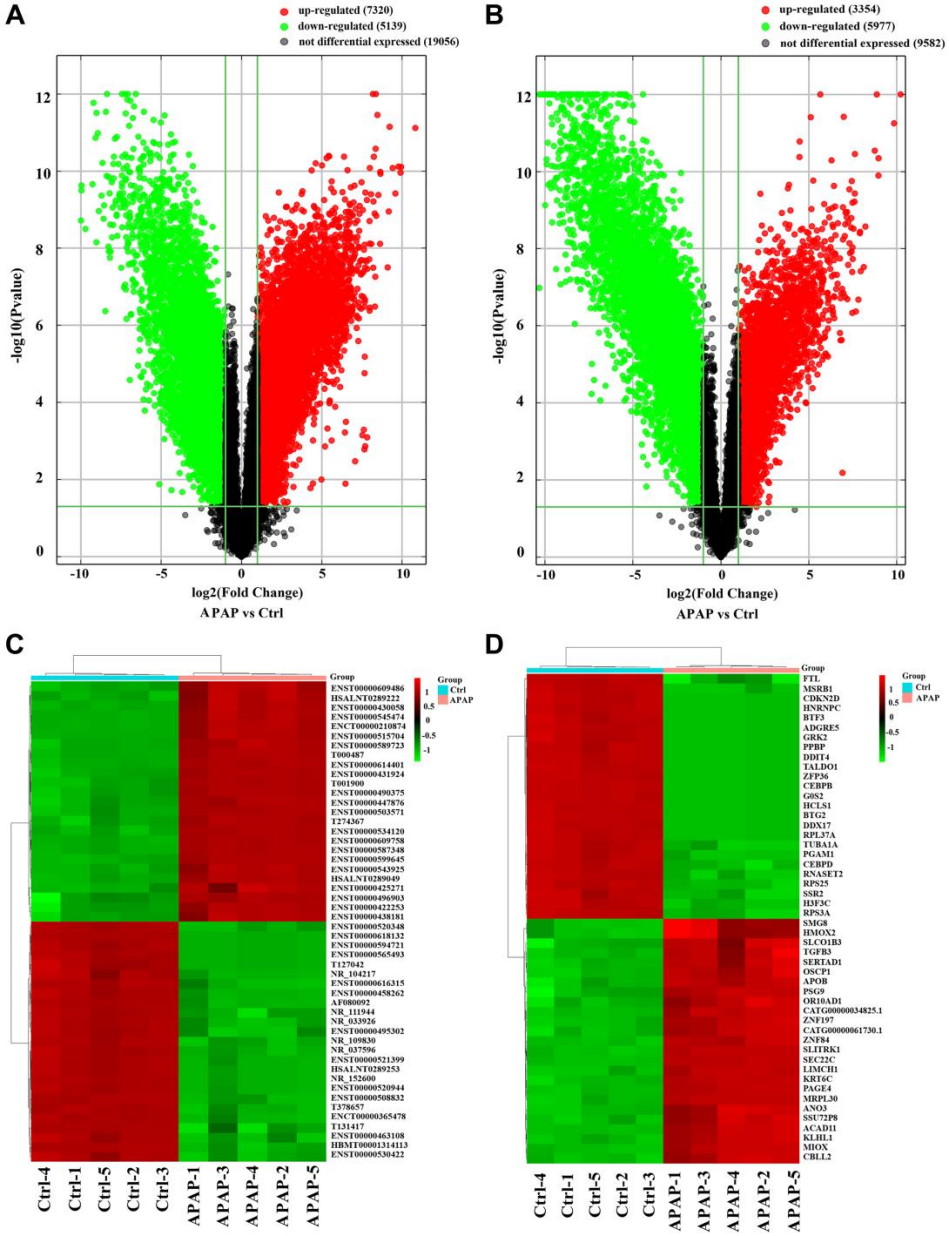
**Identification of DE lncRNAs and mRNAs in APAP patients.** (**A**, **B**) Volcano plots presenting differences in the expression of lncRNAs and mRNAs between the APAP and control groups. Values plotted on the x- and y-axes represent the averaged normalized signal values of each group (log2-scaled). Red indicates upregulation, green indicates downregulation and black indicates no difference. (**C**, **D**) Heatmaps showing the expression profiles of the top DE lncRNAs and mRNAs.

Total RNA was extracted from peripheral blood samples from APAP patients and healthy controls for lncRNA and mRNA microarray analyses. When differential expression was defined as a fold-change ≥ 2.0 and a *P*-value < 0.05, 12459 DE lncRNAs (7320 upregulated, 5139 downregulated) and 9331 DE mRNAs (3354 upregulated, 5977 downregulated) were identified in the APAP group compared with the control group. Volcano plots were used to visualize the DE lncRNAs and DE mRNAs (Figure 1A and 1B). Hierarchical clustering heatmaps of the top 50 DE lncRNAs and top 50 DE mRNAs based on their fold-changes are shown in Figure 1C and 1D, respectively. The top 10 upregulated and downregulated DE lncRNAs and the top 10 upregulated and downregulated DE mRNAs are listed in Supplementary Tables 1 and 2, respectively.

### Biological function analyses of DE mRNAs

**Figure 2:**
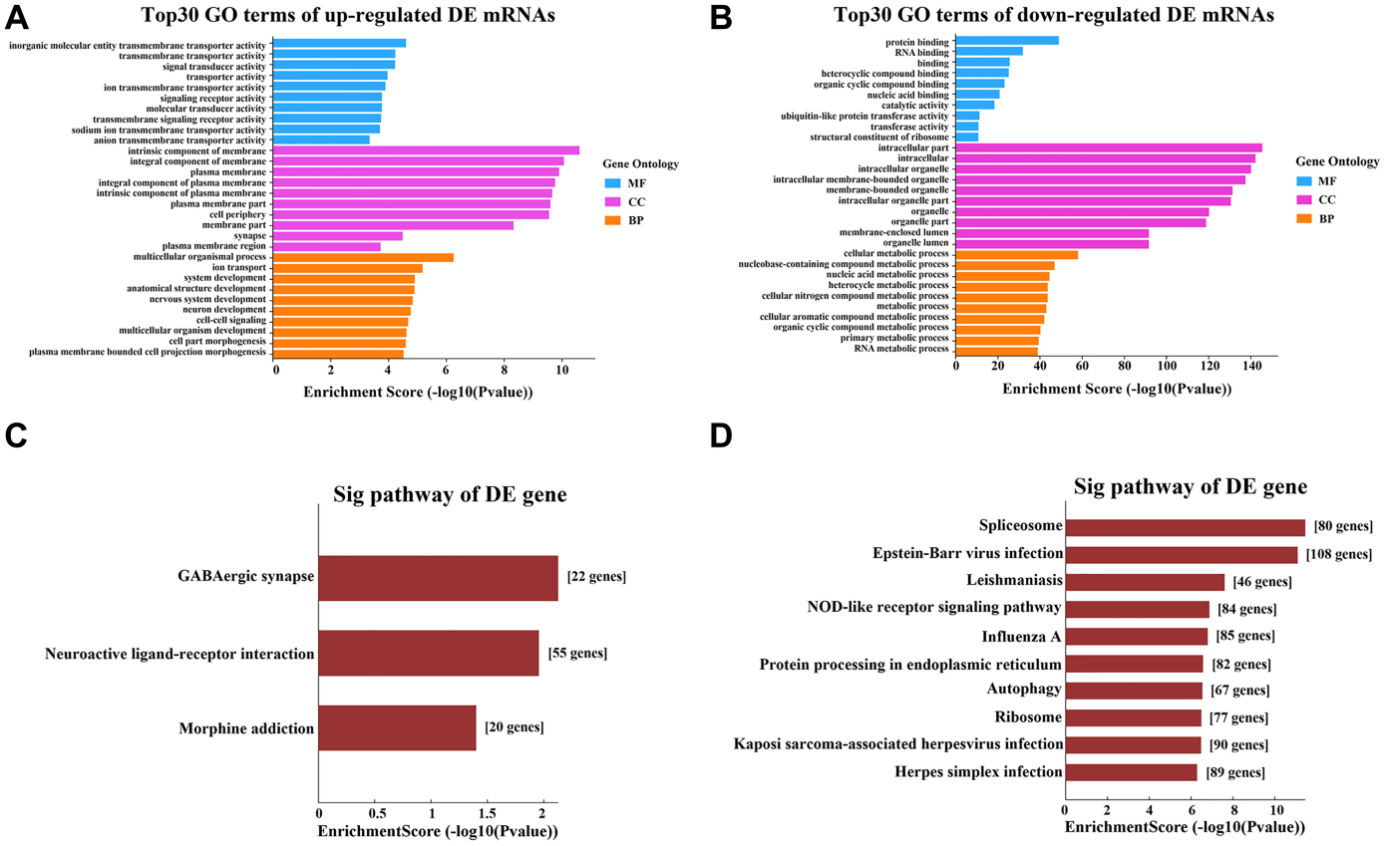
**GO and KEGG pathway analyses of the DE mRNAs.** (**A**) Top 10 terms from the GO analysis of upregulated mRNAs. (**B**) Top 10 terms from the GO analysis of downregulated mRNAs. (**C**) Upregulated mRNAs were clustered through a KEGG analysis. (**D**) Downregulated mRNAs were clustered through a KEGG analysis.

Gene Ontology (GO) analyses were used to determine the functions of the DE mRNAs in APAP patients. GO analyses categorize potential functions according to three defined terms: biological process (BP), molecular function (MF) and cellular component (CC). The top 10 BPs, CCs and MFs based on their *P*-values are shown as a bar plot in Figure 2A and 2B. GO analysis of the upregulated DE mRNAs revealed that the most enriched GO terms were “multicellular organismal process” (BP), “ion transport” (BP), “intrinsic component of membrane” (CC), “integral component of membrane” (CC), “inorganic molecular entity transmembrane transporter activity” (MF) and “transmembrane transporter activity” (MF). Among the downregulated DE mRNAs, “cellular metabolic process” (BP), “nucleobase-containing compound metabolic process” (BP), “intracellular part” (CC), “intracellular” (CC), “protein binding” (MF), and “RNA binding” (MF) had the highest enrichment scores (Figure 2A and 2B).

Kyoto Encyclopedia of Genes and Genomes (KEGG) pathway analyses were used to explore the pathways involved in APAP. Three pathways were enriched among the upregulated DE mRNAs in the APAP group: “GABAergic synapse”, “neuroactive ligand-receptor interaction” and “morphine addiction” (Figure 2C). Ninety-eight pathway terms were significantly enriched among the downregulated DE mRNAs, the top three of which were “spliceosome”, “Epstein-Barr virus infection” and “Leishmaniasis”. The top 10 KEGG pathways of the downregulated mRNAs are shown in Figure 2D.

**Figure 3:**
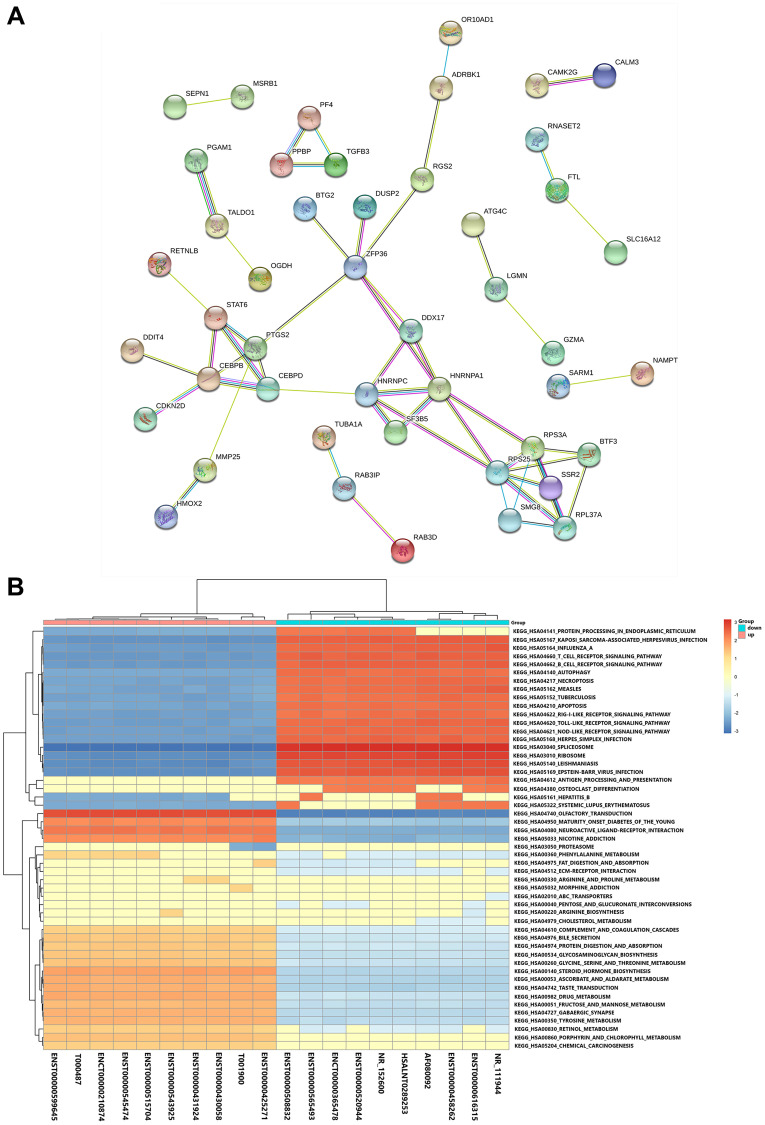
**Functional and pathway analyses.** (**A**) Protein-protein interaction network of the DE mRNAs. (**B**) GSEA of the top 10 DE lncRNAs based on their fold-changes.

In order to identify relationships among the products of the identified DE mRNAs, we constructed a protein-protein interaction network using the Search Tool for the Retrieval of Interacting Genes (STRING) database. As shown in Figure 3A, complex interactions were detected among the various DE mRNAs. We performed a gene set enrichment analysis (GSEA) to identify lncRNA-related pathways. A cluster profiler GSEA was performed on the top 10 upregulated and downregulated DE lncRNAs; the results are shown in Figure 3B.

### Quantitative real-time PCR (qRT-PCR) validation of DE lncRNAs and mRNAs

**Figure 4:**
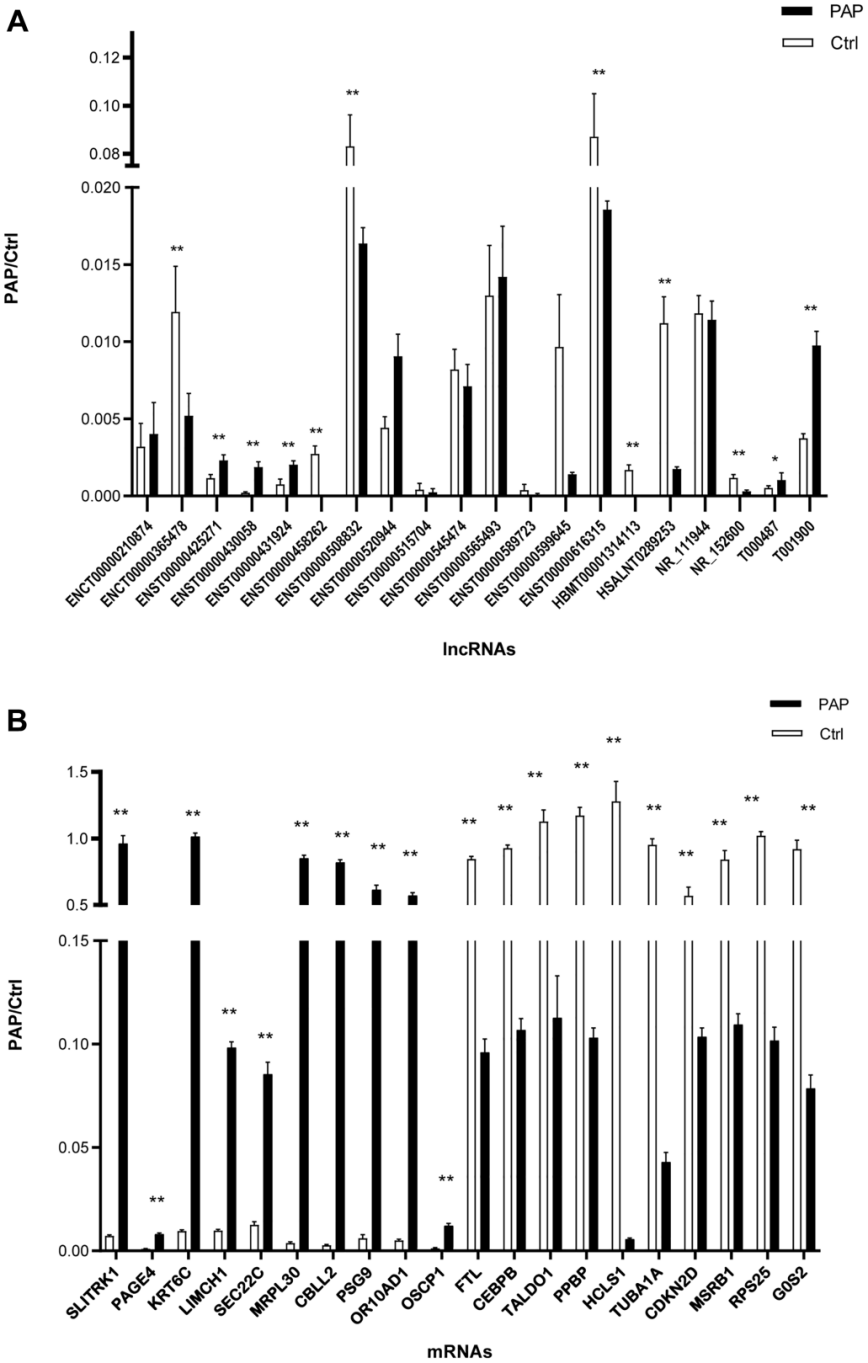
**Validation of DE lncRNAs and mRNAs.** (**A**) DE lncRNAs were confirmed using qRT-PCR. (**B**) DE mRNAs were confirmed using qRT-PCR. *N* = 5/group, ^*^P < 0.05, ^**^P < 0.01.

We then performed a qRT-PCR validation on the top 20 DE lncRNAs and 20 DE mRNAs based on their fold-changes. The qRT-PCR results were consistent with the microarray analysis results for 12 of the lncRNAs, of which five were upregulated (ENST00000425271, ENST00000430058, ENST00000431924, T000487, T001900) and seven were downregulated (ENCT00000365478, ENST00000458262, ENST00000508832, ENST00000616315, HBMT00001314113, HSALNT0289253, NR_152600). Moreover, the qRT-PCR results were consistent with the microarray analysis results for all 20 mRNAs, of which ten were upregulated (SLITRK1, PAGE4, KRT6C, LIMCH1, SEC22C, MRPL30, CBLL2, PSG9, OR10AD1, OSCP1) and ten were downregulated (FTL, CEBPB, TALDO1, PPBP, HCLS1, TUBA1A, CDKN2D, MSRB1, RPS25, G0S2) (Figure 4A and 4B).

### Coding and noncoding co-expression (CNC) network analysis

**Figure 5:**
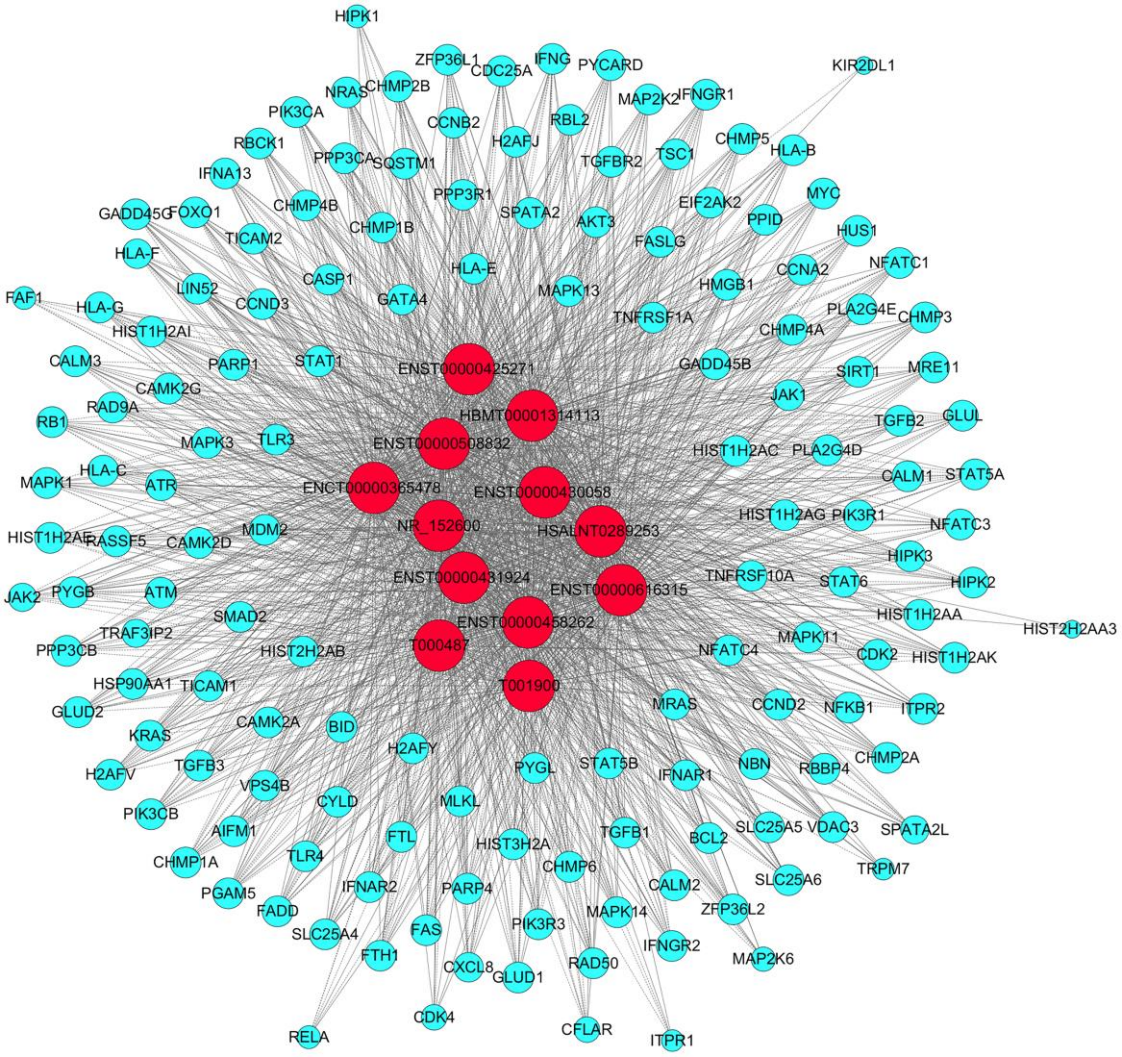
**CNC network analysis.** Red nodes are lncRNAs; blue nodes are mRNAs. Positive correlations are represented by solid lines; negative correlations are represented by dashed lines.

To determine the potential regulatory relationships between the lncRNAs and mRNAs, we calculated the correlation coefficients between the normalized expression data of the 12 validated DE lncRNAs and all 9331 DE mRNAs. Those with Pearson’s correlation coefficients > 0.95, *P*-values ≤ 0.05 and false discovery rates ≤ 1 were used to construct a CNC network. ENCT00000365478 correlated with 147 mRNAs, ENST00000425271 with 146 mRNAs, ENST00000430058 with 142 mRNAs, ENST00000431924 with 147 mRNAs, ENST00000458262 with 147 mRNAs, ENST00000508832 with 146 mRNAs, ENST00000616315 with 147 mRNAs, HBMT00001314113 with 147 mRNAs, HSALNT0289253 with 149 mRNAs, NR_152600 with 149 mRNAs, T000487 with 148 mRNAs, and T001900 with 148 mRNAs. The CNC network plot is shown in Figure 5.

**Figure 6:**
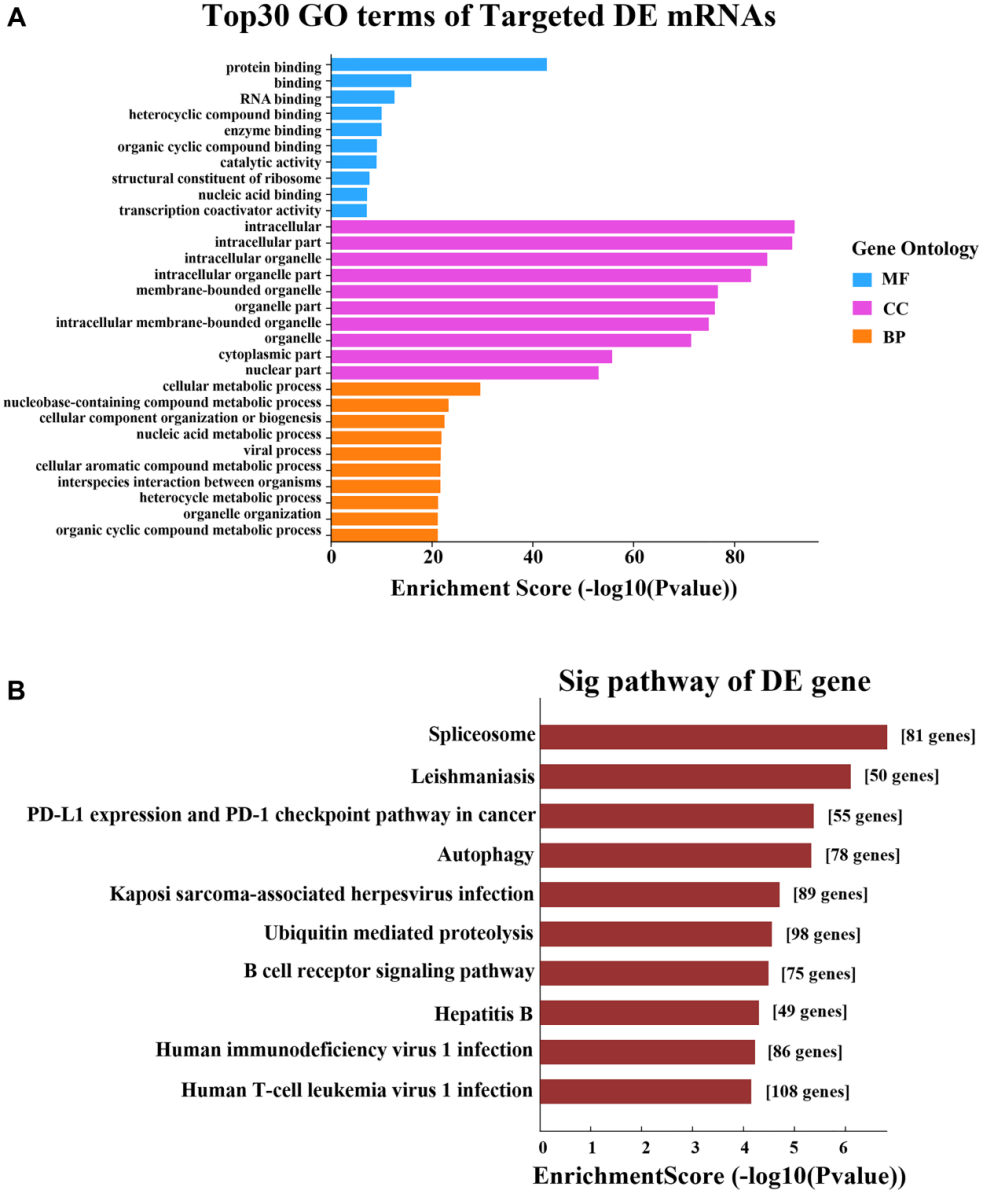
**GO and KEGG pathway analyses based on the CNC network.** (**A**) GO analysis. (**B**) KEGG pathway analysis.

Then, we performed GO and KEGG analyses on the predicted target mRNAs in the network. The GO analysis indicated that the top two enriched BPs were “cellular metabolic process” and “nucleobase-containing compound metabolic process”, the top two enriched CCs were “intracellular” and “intracellular part”, and the top two enriched MFs were “protein binding” and “binding” (Figure 6A). The KEGG analysis revealed a total of 81 enriched pathways, of which the main pathways were “spliceosome”, “leishmaniasis”, “PD-L1 expression” and “PD-1 checkpoint pathway in cancer” (Figure 6B).

### Competing endogenous RNA (ceRNA) network analysis

**Figure 7:**
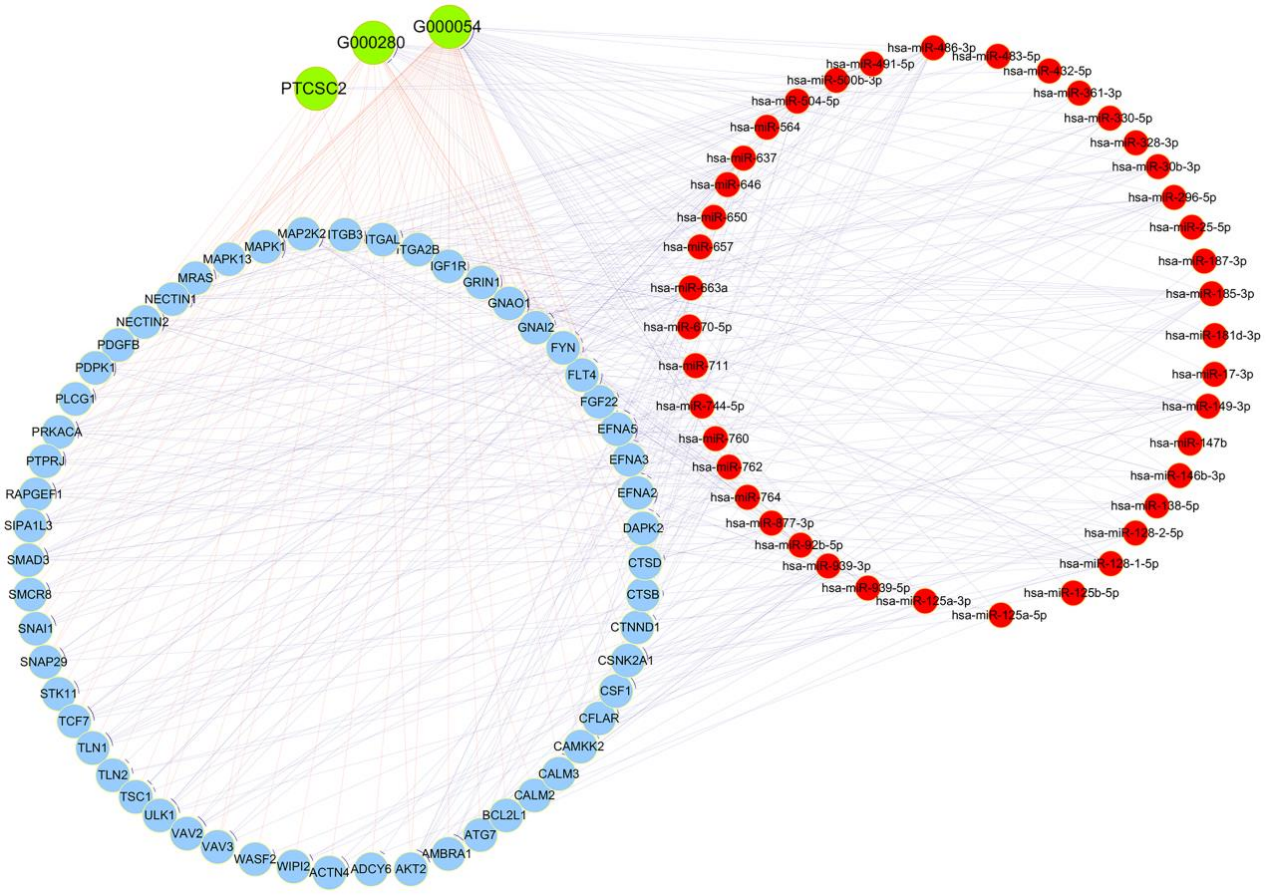
**CeRNA network analysis.** Red circles represent miRNAs, blue circles represent mRNAs and green circles represent lncRNAs.

The ceRNA theory posits that lncRNAs can sponge miRNAs and thus derepress miRNA-inhibited mRNAs. To determine whether the DE lncRNAs in this study were involved in ceRNA networks, we constructed a co-expression network of lncRNA-miRNA-mRNA interactions using all the DE mRNAs and the 12 DE lncRNAs validated by qRT-PCR. The number of predicted miRNA IDs was confined to 1000, and the predicted target genes of these miRNAs were subjected to KEGG and GO analyses. The KEGG analysis revealed a total of 50 enriched pathways, from which we selected three pathways associated with the research background: “Adherens junction - Homo sapiens”, “Rap1 signaling pathway - Homo sapiens” and “Autophagy - animal - Homo sapiens (human)” to construct the ceRNA network. The DE mRNAs in these pathways were used to construct the ceRNA network (Figure 7).

**Figure 8:**
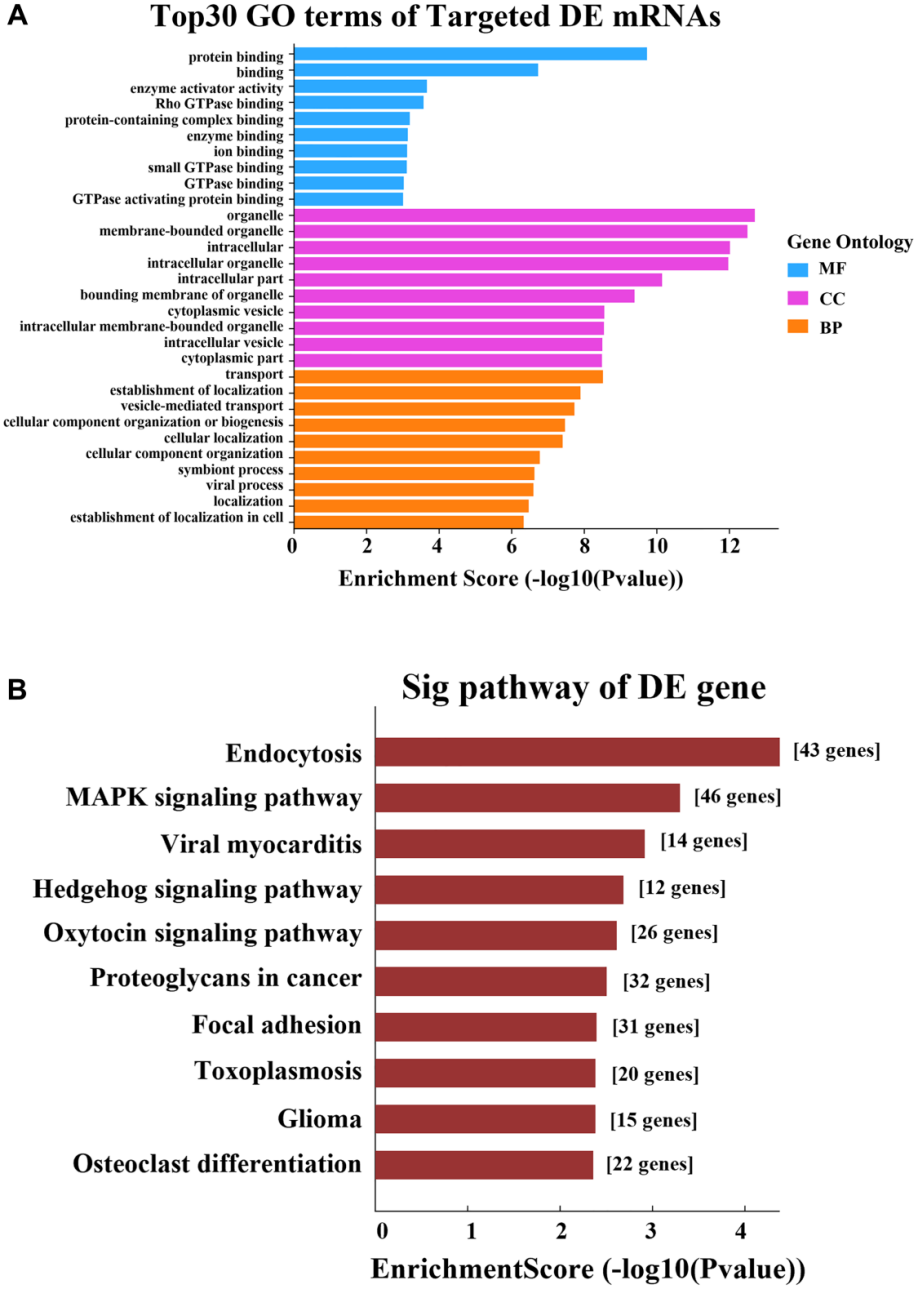
**GO and KEGG pathway analyses based on the ceRNA network.** (**A**) GO analysis. (**B**) KEGG pathway analysis.

In the GO analysis, the top two enriched BPs were “transport” and “establishment of localization”, the top two enriched CCs were “organelle” and “membrane-bounded organelle”, and the top two enriched MFs were “protein binding” and “binding” (Figure 8A). The KEGG pathway analysis revealed that the main pathways were “endocytosis”, “MAPK signaling pathway” and “viral myocarditis” (Figure 8B).

## DISCUSSION

LncRNAs are associated with a variety of autoimmune diseases, but there have been relatively few studies on lncRNA expression patterns in human APAP. In the present study, we compared the lncRNA and mRNA expression profiles of peripheral blood samples from five APAP patients and five matched healthy controls. In total, 7320 lncRNAs were upregulated and 5139 lncRNAs were downregulated in the APAP group compared with the control group. In addition, 3354 mRNAs were upregulated and 5977 mRNAs were downregulated in the APAP group. Thus, the expression patterns of lncRNAs and mRNAs differed significantly between APAP patients and healthy controls. We then performed in-depth bioinformatics analyses using the top 12 qRT-PCR-validated DE lncRNAs, and constructed CNC and ceRNA networks from these 12 DE lncRNAs and all the DE mRNAs. The 12 lncRNAs were ENST00000425271, ENST00000430058, ENST00000431924, T000487, T001900, ENCT00000365478, ENST00000458262, ENST00000508832, ENST00000616315, HBMT00001314113, HSALNT0289253 and NR_152600.

Our KEGG analysis of upregulated DE mRNAs revealed that the most enriched pathways related to APAP were “GABAergic synapse”, “neuroactive ligand-receptor interaction” and “morphine addiction”. These results suggested that changes in neurotransmitter signaling also occur in the blood during APAP. Neurotransmitter signal transduction usually impacts the nervous system, but many neurotransmitters also influence the cells of the immune system [12]. GABA (γ-aminobutyric acid) is the main inhibitory neurotransmitter in the brain, but is also present in the pancreatic islets and the bloodstream [13]. Immune cells in the blood express GABA receptors [14], and GABA regulates the release of various cytokines from peripheral blood mononuclear cells and CD4+ T cells in a concentration-dependent manner. In peripheral blood mononuclear cells from type I diabetic patients, GABA was found to alter the secretion of both pro- and anti-inflammatory cytokines [15]. However, neurotransmitter signaling in APAP has not previously been explored. GM-CSF is secreted by cells of the adaptive immune system (T cells and B cells) and bone marrow (monocytes, macrophages, mast cells and neutrophils). Since APAP is generally linked to autoantibodies against GM-CSF, we speculated that GABA regulation of immune cells might promote the production of these antibodies. Our results have provided a new direction for future in-depth research.

We also performed a GO analysis on the downregulated mRNAs in APAP patients, which revealed significant enrichment of “ubiquitin-like protein transferase activity”, a MF (GO:0019787). We then performed a CNC analysis to investigate the downstream genes of the top 12 DE lncRNAs, and conducted a GO analysis on these predicted target genes. “Ubiquitin-like protein transferase activity” was also significantly enriched in this analysis, indicating that this MF was significantly downregulated in APAP patients in a manner dependent on the 12 validated lncRNAs. Small proteins such as ubiquitin and ubiquitin-like proteins are crucial post-translational modifiers of eukaryotic proteins [16, 17]. The ubiquitin-like proteins, including members of the small ubiquitin-related modifier family, interferon-stimulated gene 15 and HLA-F-adjacent transcript 10, have the same three-dimensional folding characteristics as ubiquitin, but are quite different in other respects [18]. Ubiquitin-like protein modifications alter the conformation, subcellular localization, stability and binding affinity of their targets, thereby influencing cellular activities such as transcription, proliferation, differentiation, proteolysis, signal transduction, autophagy, protein synthesis and antiviral responses [19]. Alterations in ubiquitin-like protein pathways have been implicated in cardiovascular diseases, viral defenses, different types of cancer and neurodegenerative diseases [20–22].

Increasing recognition of the importance of ubiquitin-like protein conjugation pathways in various diseases has prompted intensive research into selective inhibitors of these pathways [23]. Bortezomib, a specific inhibitor of the chymotrypsin activity of the proteasome, was approved by the Food and Drug Administration in 2008 as the first proteasome inhibitor for the treatment of multiple myeloma [24, 25]. Small molecule inhibitors of enzymes in ubiquitin-like protein conjugation pathways have also been developed to treat infectious diseases, cancer, immuno-inflammatory disorders, neurodegenerative disorders and cardiovascular disease. Our study suggested that ubiquitin-like protein transferases could be a new therapeutic target in APAP, and that their expression could be modified via treatments that alter the levels of the 12 lncRNAs validated in this study. Thus, in-depth studies of these 12 lncRNAs are warranted.

LncRNAs upregulate mRNA expression by sponging miRNAs [26]. In our KEGG analysis based on the ceRNA network, the “MAPK signaling pathway” was significantly enriched, indicating that our 12 validated lncRNAs regulate the “MAPK signaling pathway” by sponging certain miRNAs. The MAPK (mitogen-activated protein kinase) signaling pathway regulates the expression of pulmonary surfactant proteins (SPs). As H_2_O_2_ induces oxidative stress, the MAPK and signal transducer and activator of transcription pathways prevent thyroid transcription factor 1 from binding to DNA, thereby regulating the expression of *SP-A* and *SP-B* [27]. In sepsis-induced multiple organ injury, SP-A and SP-D were found to attenuate lipopolysaccharide-induced apoptosis, possibly by preventing lipopolysaccharide from activating the p38 MAPK pathway [28].

Pulmonary surfactants are important for surfactant metabolism and pulmonary innate immunity, and their expression is regulated by noncoding RNAs. After SP-A knockout mice were exposed to O_3_-induced oxidative stress, 24 miRNAs were differentially expressed, including regulators of MAPK signaling, the cell cycle, anti-apoptotic activity, etc. [29]. In APAP, surfactant deposition is mainly due to reduced clearance, but abnormal surfactant production may also occur. Our study suggested that 12 DE lncRNAs regulate the “MAPK signaling pathway” by sponging certain miRNAs, and thus might regulate surfactant expression in APAP.

Our GO analysis revealed that the upregulated mRNAs in the APAP group were involved in many transmembrane ion transport processes. The significantly enriched MFs included “inorganic molecular entity transmembrane transporter activity”, “transmembrane transporter activity”, “ion transmembrane transporter activity”, “transmembrane signaling receptor activity”, “sodium ion transmembrane transporter activity” and “anion transmembrane transporter activity”. These findings suggested that abnormalities in ion transport are involved in APAP, a prospect worthy of further study.

Generally, APAP is considered to be an autoimmune disease characterized by high serum and lung levels of GM-CSF autoantibodies, which prevent alveolar macrophages from clearing pulmonary surfactants. Interestingly, our microarray results did not reveal any significant changes in immune processes, possibly because the mean GM-CSF antibody level in our APAP group (110.44 ng/mL) was significantly lower than the levels reported in previous studies of APAP patients (40.5 μg/mL [30], 66.8 ± 71.7 μg/mL [5] and 102 μg/mL [31]). For this reason, we also did not identify any lncRNA molecules involved in immune processes. Thus, although APAP patients produce autoantibodies against GM-CSF, APAP may proceed by mechanisms other than immune abnormalities, which should be studied in depth.

In conclusion, our study revealed for the first time that “ubiquitin-like protein transferase activity” is reduced in APAP patients and regulated by lncRNAs, and that the “MAPK signaling pathway” is regulated by ceRNAs. These pathways then alter surfactant expression, and thus could be therapeutic targets in APAP. Importantly, we also identified 12 significantly DE lncRNAs as candidate genes that are dysregulated in APAP. Transmembrane ion transport is an additional direction worthy of study. Our data had certain limitations, as the sample size was insufficient for sequencing. Nevertheless, this study has provided a foundation for future research on the involvement of lncRNAs in APAP, and has revealed potential therapeutic targets for this disease.

## MATERIALS AND METHODS

### Patients and sample collection

Peripheral blood samples from five adults with APAP and five healthy volunteers were obtained at Peking Union Medical College Hospital between November and December of 2019. All the APAP cases were clinically diagnosed according to published APAP diagnosis criteria [32]. Patients diagnosed with other types of pulmonary alveolar proteinosis were excluded. The control subjects were healthy volunteers matched to the APAP cases on the basis of age and gender. The baseline demographic summary of the APAP and control groups is shown in Table 1. The characteristics of the APAP patients are listed in Table 2. This study was approved by the Peking Union Medical College Hospital Ethics Committee, and each patient provided signed informed consent for research purposes (No. JS-1233).

**Table 1.** Demographic characteristics between APAP patients and control.

**Characteristics**	**APAP *N* = 5**	**Control *N* = 5**	P^*^
Gender, male, *n* (%)	2 (40)	2 (40)	1.000
Age	36 (21–49)	34 (25–45)	0.917
Smoking history, *n* (%)	2 (40)	0 (0)	0.444
Dust inhalation, *n* (%)	0 (0)	0 (0)	1.000

^*^P values are for comparison between APAP and control group. The *P* value was calculated with the use of the Mann-Whitney U for compare continuous variables. The *P* value was calculated with the use of Fisher Chi-squared test for categorical variables.

Abbreviations: APAP: autoimmune pulmonary alveolar proteinosis.

**Table 2.** Characteristics of patients with APAP.

**Patients**	**Gender**	**Age year**	**PO_2_ (mmHg)**	**D(A-a) O_2_ (mmHg)**	**CEA U/L**	**LDH U/L**	CT^*^ %	**6WMD (m)**	Brog^**^	**SGRQ total**	**Anti GM-CSF Ab ng/ml**	**FEV_1_ %pred**	**FVC %pred**	**DLCO %pred**
1#	F	34	71	41.5	7.3	373	>80	475	2	26	37.8	NA	NA	NA
2#	F	27	79	30.7	4.4	142	30–50	460	3	57	100.09	79.8	79	46.89
3#	M	25	58	56.6	NA	468	>80	468	2	53	23.6	63.8	61.1	26.76
4#	F	39	88	18.2	1.55	152	10–30	610	0	13	23.63	99.5	106.4	81.41
5#	M	45	73	39.8	NA	471	50–80	310	0	51	146.2	86.8	86.1	39.65

^*^The severity of abnormalities was graded according to the percentage of the volume judged abnormal.

^**^Borg score after exercise.

Abbreviations: Anti-GM-CSF Ab: Anti granulocyte-macrophage colony-stimulating factor antibody; APAP: autoimmune pulmonary alveolar proteinosis; CEA: carcinoembryonic antigen; CT: computed tomography; DLCO: carbon monoxide diffusion capacity; FEV_1_: forced expiratory volume in 1 second; FVC: forced vital capacity; LDH: lactate dehydrogenase; SGRQ: St. George Respiratory Questionnaire; 6MWD: 6-min walking distance.

### RNA labeling and array hybridization

RNA quantity and quality were measured on a NanoDrop ND-1000 (NanoDrop, USA). RNA integrity was assessed using standard denaturing agarose gel electrophoresis or an Agilent 2100 Bioanalyzer. Sample labeling and array hybridization were performed according to the Agilent One-Color Microarray-Based Gene Expression Analysis protocol (Agilent Technologies, USA) with minor modifications. Briefly, mRNA was purified from total RNA after the removal of rRNA (mRNA-ONLY™ Eukaryotic mRNA Isolation Kit, Epicentre, USA). Then, each sample was amplified and transcribed into fluorescent complementary RNA (cRNA) along the entire length of the transcript without 3' bias using a random priming method (Arraystar Flash RNA Labeling Kit, Arraystar, USA). The labeled cRNAs were purified with an RNeasy Mini Kit (Qiagen, German). The concentrations and specific activities of the labeled cRNAs (pmol Cy3/μg cRNA) were measured on a NanoDrop ND-1000. Then, 1 μg of each labeled cRNA was fragmented through the addition of 5 μL of 10× Blocking Agent and 1 μL of 25× Fragmentation Buffer. The mixture was heated to 60°C for 30 min, and then 25 μL of 2× GE Hybridization buffer was added to dilute the labeled cRNA. In total, 50 μL of the hybridization solution was dispensed into a gasket slide and assembled on a lncRNA expression microarray slide. The slides were incubated for 17 hours at 65°C in an Agilent Hybridization Oven. The hybridized arrays were washed, fixed and scanned using an Agilent DNA Microarray Scanner (part number G2505C, USA) [33–35].

### Microarray analysis

The microarray hybridization and analysis were performed by KangChen Biotech (Shanghai, China). An Arraystar Human lncRNA Microarray V5.0 (Agilent, USA) was used, which can detect approximately 39,317 “Gold Standard and Reliable” lncRNAs and 21,174 coding transcripts. Arraystar maintains high-quality proprietary lncRNA transcriptome databases and collects lncRNAs through all major public databases and repositories, including FANTOM5 CAT (v1), GENECODE (v29), RefSeq (Updated to 2018.11), BIGTranscriptome (v1), knownGene (updated to 2018.11), lncRNAdb, lncRNAWiki, RNAdb, NRED, CLS FL, NONCODE (v5) and MiTranscriptome (v2), as well as through knowledge-based mining of scientific publications.

Agilent Feature Extraction software (version 11.0.1.1) was used to analyze the acquired array images. Quantile normalization and subsequent data processing were performed using the GeneSpring GX v12.1 software package (Agilent Technologies, USA). After quantile normalization of the raw data, lncRNAs and mRNAs that were flagged as Present or Marginal (“All Targets Value”) in at least 5 out of 10 samples were chosen for further analysis. DE lncRNAs and mRNAs with statistical significance between the two groups were identified through *P*-value/false discovery rate filtering and fold-change filtering.

Pathway and GO analyses were used to determine the biological pathways and GO terms associated with the DE mRNAs. Hierarchical clustering and combined analyses of the top 50 terms were performed using in-house scripts. The GEO accession number of the dataset in this study is GSE153957.

### qRT-PCR analysis

The levels of selected DE lncRNAs and DE mRNAs were validated using qRT-PCR. Total RNA was extracted from peripheral blood samples using TRIzol reagent (Invitrogen Life Technologies, USA). Agarose gel electrophoresis was used to determine the RNA integrity. Then, cDNA was synthesized from 1-2 μg of RNA using a RevertAid First Strand cDNA Synthesis Kit (Promega, USA). The qRT-PCR was performed using a DyNAmo Color Flash SYBR Green qPCR Kit on a Thermo PikoReal PCR System (Thermo Fisher Scientific, USA). β-Actin was used as the internal control for lncRNA and mRNA. Relative gene expression was quantified using the 2^−ΔΔCt^ method. The primer sequences are listed in Supplementary Tables 3 and 4.

### Regulatory network analysis of DE lncRNAs and DE mRNAs

#### CNC network

A CNC network was constructed to predict the interactions among the DE lncRNAs and DE mRNAs. A hybrid hierarchical clustering algorithm was used to analyze the relationships among different genes, and a correlation coefficient was calculated for each pair. LncRNA-mRNA pairs of interest were identified based on Pearson’s correlation coefficients ≥ 0.95.

#### ceRNA network

LncRNAs and mRNAs can compete for the same miRNA response elements to derepress the targets of miRNAs. We used miRanda to predict miRNA binding seed sequence sites, and considered overlapping miRNA binding sites on lncRNAs and mRNAs to represent lncRNA-miRNA-mRNA interactions. TargetScan (http://www.targetscan.org/vert_71/) was used for analysis. The ceRNA network was constructed and illustrated using Cytoscape (v3.4.0).

#### Bioinformatics analysis

The potential functions of the DE lncRNAs were analyzed through GO (http://www.geneontology.org) and KEGG (https://www.genome.jp/kegg/) analyses. The GO analysis was used to illustrate the unique biological significance of the DE genes. The KEGG analysis was performed to identify crucial pathways related to gene maps based on the latest KEGG database. Fisher’s exact test was used, with lower *P-*values indicating greater pathway significance (the cutoff *P-*value was 0.05).

The relationships among key target genes were also identified using a protein-protein interaction network, which was constructed using the STRING database (https://string-db.org/). A GSEA Java program was used for functional pathway enrichment analysis. GSEA was used to identify KEGG pathways enriched in the top 10 DE lncRNAs. A GSEA-based pathway enrichment analysis was performed on each lncRNA countergene in the lncRNA-mRNA network. The GSEA results were searched for pathways with adjusted *P*-values < 0.05.

### Statistical analysis

Statistical analyses were performed using GraphPad Prism 5 (GraphPad Software, USA). Student's *t*-test (Mann-Whitney U) was used to determine the differences between two groups. All tests were two-sided. *P*-values < 0.05 were considered statistically significant.

### Data availability statement

The data that support the findings of this study are openly available in the GenBank database under accession number GSE153957. (https://www.ncbi.nlm.nih.gov/geo/query/acc.cgi?acc=GSE153957).

## Supplementary Material

Supplementary Tables
