# Supplementary material for: Expression profiles and potential functions of long noncoding RNAs and mRNAs in autoimmune pulmonary alveolar proteinosis patients
Source: Aging (Albany NY). 2021 Apr 4;13(7):10535–54. doi: 10.18632/aging.202818 (PMC8064141; doi:10.18632/aging.202818)
Supplement: Supplementary Tables [file aging-13-202818-s001.pdf]

SUPPLEMENTARY MATERIALS

Supplementary Tables

Supplementary Table 1. The detailed information of the top ten up-regulated and top ten down-regulated lncRNAs.

Probe Name	*P*-value	Fold Change	Regulation	Transcript_ID	Gene Symbol	chrom	strand	RNA length
ASHG19LNC1A100068731V5	7.6E-12	1818.40313	up	ENST00000599645	AC007193.1	chr19	-	448
ASHGV40004606V5	7.81E-11	982.901625	up	ENST00000545474	AC018410.1	chr11	-	492
ASHGV40024521V5	1.098E-10	959.513477	up	T001900	G000280	chr1	+	1549
ASHG19LNC1A105234326V5	7.66E-11	877.701206	up	ENST00000515704	LINC02208	chr5	-	718
ASHGV40004607V5	3.806E-10	768.098116	up	ENST00000543925	AC018410.1	chr11	-	422
ASHGV40000720V5	8.3E-11	683.543035	up	ENST00000431924	AL021707.2	chr22	-	786
ASHGV40057877V5	7.1E-12	599.638432	up	T000487	G000054	chr1	-	3599
ASHGV40052067V5	1.1146E-09	576.51895	up	ENST00000430058	PTCSC2	chr9	-	465
ASHGV40041255V5	3.1497E-08	551.050383	up	ENST00000425271	AL390115.1	chr1	+	423
ASHG19LNC1A105269571V5	1.169E-10	403.963558	up	ENCT00000210874	CATG00000040186.1	chr19	-	10619
ASHG19LNC1A100057232V5	1.913E-09	1016.27787	down	ENCT00000365478	CATG00000082103.1	chr5	-	390
ASHG19LNC1A110525699V5	3.167E-10	997.248819	down	ENST00000508832	MALAT1	chr11	+	1519
ASHGV40008645V5	2.36E-10	995.399588	down	AF080092	AF080092	chr11	+	481
ASHG19LNC1A100474151V5	3.1635E-09	839.872372	down	ENST00000616315	NEAT1	chr11	+	512
ASHG19LNC1A107196674V5	2.186E-10	619.007595	down	ENST00000458262	RP11-640M9.1	chr1	-	521
ASHGV40030799V5	1.7E-12	592.850075	down	ENST00000565493	NORAD	chr20	-	5339
ASHGV40001710V5	1.37E-11	543.467774	down	ENST00000520944	SNHG6	chr8	-	638
ASHGV40004524V5	7.0301E-09	522.917702	down	NR_111944	RPS17	chr15	-	731
ASHG19LNC1A102452129V5	3.1E-12	520.425982	down	NR_152600	SNHG6	chr8	-	704
ASHG19LNC1ABL100000387V5	1.04E-11	492.583195	down	HSALNT0289253	SNHG6	chr8	-	476

Supplementary Table 2. The detailed information of the top ten up-regulated and top ten down-regulated mRNAs.

Probe Name	*P*-value	Fold Change	Regulation	Transcript_ID	Gene ID	Gene Symbol	chrom	strand
ASHG19AP1B106949850V5	3E-13	1192.01593	up	ENST00000377084	ENSG00000178235	SLITRK1	chr13	-
ASHG19AP1B106176451V5	5.6E-12	922.2951365	up	ENST00000218068	ENSG00000101951	PAGE4	chrX	+
ASHG19AP1B117097439V5	4.5E-11	500.1659654	up	ENST00000252250	ENSG00000170465	KRT6C	chr12	-
ASHG19AP1B126573651V5	1.266E-10	497.5281276	up	ENST00000503057	ENSG00000064042	LIMCH1	chr4	+
ASHG19AP1B123266321V5	0	466.3138857	up	ENST00000264454	ENSG00000093183	SEC22C	chr3	-
ASHG19AP1B124077268V5	2.88E-11	430.8211361	up	ENST00000338148	ENSG00000185414	MRPL30	chr2	+
ASHG19AP1B100226564V5	2.675E-09	297.697864	up	ENST00000323684	ENSG00000175809	CBLL2	chrX	+
ASHG19AP1B111519354V5	2.9981E-08	279.205045	up	ENST00000291752	ENSG00000183668	PSG9	chr19	-
ASHG19AP1B100197595V5	3.83093E-08	247.851366	up	ENST00000310248	ENSG00000172640	OR10AD1	chr12	-
ASHG19AP1B125673864V5	3.6512E-09	241.3310941	up	ENST00000356637	ENSG00000116885	OSCP1	chr1	-
ASHG19AP1B100220364V5	1.04435E-07	1282.01766	down	ENST00000331825	ENSG00000087086	FTL	chr19	+
ASHG19AP1B100188143V5	0	1275.723095	down	ENST00000303004	ENSG00000172216	CEBPB	chr20	+
ASHG19AP1B109215451V5	4E-13	1189.684161	down	ENST00000319006	ENSG00000177156	TALDO1	chr11	+
ASHG19AP1B100053808V5	4E-13	1073.782215	down	ENST00000296028	ENSG00000163736	PPBP	chr4	-
ASHG19AP1B100093135V5	0	1048.517409	down	ENST00000314583	ENSG00000180353	HCLS1	chr3	-
ASHG19AP1B122794202V5	7.459E-10	1014.582271	down	ENST00000301071	ENSG00000167552	TUBA1A	chr12	-
ASHG19AP1B116719216V5	6E-13	973.5239086	down	ENST00000393599	ENSG00000129355	CDKN2D	chr19	-
ASHG19AP1B100140381V5	4.83E-11	961.08428	down	ENST00000334828	ENSG00000171314	PGAM1	chr10	+
ASHG19AP1B105936045V5	6.47E-11	953.1382382	down	ENST00000361871	ENSG00000198736	MSRB1	chr16	-
ASHG19AP1B123464220V5	1.79E-11	915.3471079	down	ENST00000527673	ENSG00000118181	RPS25	chr11	-

Supplementary Table 3. Primers for lncRNAs validated by qRT-PCR.

Gene	Forward and Reverse primer sequence
β-actin（H）	F:5′ GTGGCCGAGGACTTTGATTG3′<br>R:5′ CCTGTAACAACGCATCTCATATT3′
ENST00000599645	F:5′ GCATATTTGTCAATGTTTCCAG 3′<br>R:5′ TCGTCAGACCTACAGAGAGCTTA 3′
ENST00000545474	F:5′ CTTCTCCCTCATCTGTCTGTAG 3′<br>R:5′ ATGGCGTGCTCAGAAACC 3′
ENST00000515704	F:5′ ATTGAGCATCCCTGACCC 3′<br>R:5′ ACTGAGAACTCCAAAATGTGAT 3′
ENST00000431924	F:5′ GTCTGTCTCATGCACTTGCTC 3′<br>R:5′ TTCTGGAGACTGGGAAGTCC 3′
ENST00000430058	F:5′ CGAACTGGATATAGGTATGAGAGAA 3′<br>R:5′ GGCAGAAAGGAGGTTTGGTC 3′
ENST00000425271	F:5' GCTCCGTTAGTGACCTGCTT 3′<br>R:5′ TGATCGTTAATGAGAACTTCCTC 3′
ENST00000508832	F:5′ TTTGTATTATCAAACTTTTTTCAGA 3′<br>R:5′ AGTAAGAATCTCAGGGTTATGCT 3′
ENST00000616315	F:5′ GTATGTAAATCGTGCCTTTACTAC 3′<br>R:5′ ATAAAAAGCCATTGGTATTACTTT 3′
ENST00000458262	F:5′ GTGTTCAAGGCAAGACCGA 3′<br>R:5′ TTCCTATCCATCCCCTCAGTA 3′
ENST00000565493	F:5′ CAGCGCAGAGAACTGCCAAG 3′<br>R:5′ TGGGAAAGAGAGGTTCGCTG 3′
ENST00000520944	F:5′ CTTCCGATGTCGCTCTTCCT 3′<br>R:5′ ACCACACCGGCATGACTAAC 3′
NR_111944	F:5′ CCATTTCCAGGACTTCGG 3′<br>R:5′ AATTCATCCCAACTGTAGGCT 3′
NR_152600	F:5′ ACGCGGCATGTATTGAGGTT 3′<br>R:5′ ACACTTGAGGTAACGAAGCAGAA 3′
T001900	F:5′ ACGATGGAGTATTCTGGCTGTT 3′<br>R:5′ CAGTCATGTAACGAGAGTGGGAT 3′
T000487	F:5′ CCTGGTTGCAGATGGAAAAGAT 3′<br>R:5′ AGTCAACAAGAACAGGAACTTTGTC 3′
HSALNT0289253	F:5′ GCGGCATGTATTGAGGCATA 3′<br>R:5′ GCCACACCTTGAGGTAACGA 3′
ENCT00000210874	F:5′ GGGCTTATGGGTGTCCTA 3′<br>R:5′ ATACGGGTGCTTCTGGTT 3′
ENCT00000365478	F:5' GACTGGTTACACGATCCCTGAG 3′<br>R:5′ GAGCGTCACAGACCTGTTATTG 3′
ENST00000589723	F:5′ GAGCAGTCAAGGAAGTTTTGGAAG 3′<br>R:5′ CTGGTTACCCCGAGACTCGTGT 3′
HBMT00001314113	F:5′ AACTGAAGAAATTCATGACGCG 3′<br>R:5′ GTTCCCTTGGCTGTGGTTTT 3′

Supplementary Table 4. Primers for mRNAs validated by qRT-PCR.

Gene	Forward and Reverse primer sequence
β-actin	F:5′GTGGCCGAGGACTTTGATTG3′<br>R :5′ CCTGTAACAACGCATCTCATATT3′
SLITRK1	F:5′ GGGCAACAATAACATCGCTAC3′<br>R:5′ CGTTGTACTCCACGTTCAGGT3′
PAGE4	F:5′ CAGCAAGAGGAACCACCAACT3′<br>R:5′ CCTGGCAATCACCTTCTACTTTAC3′
KRT6C	F:5′ CTGGGGCTTATTTCAGAACAACT3′<br>R:5′ GAGAAATCATCACAGGGAGAAAGA3′
LIMCH1	F:5′ AGAACGCAGATACTATGAGGAGG3′<br>R:5′ CAGCGGAACTTGAAGAAACAG3′
SEC22C	F:5′ TCTCAGATGGAGTGCAGCTTG3′<br>R:5′ ACACATGATGTTGAGAATGAGGG3′
MRPL30	F:5′ TGCGCTTAGTAGTTCAATGGC3′<br>R:5′ CAGTTTATGAGGGTTCTGTGGA3′
CBLL2	F:5′ TGAGGCGCATAAACGAGGTT3′<br>R:5′ GCGGAGCAATATGAGGACGA3′
PSG9	F:5′ TGACTGCAACAACTGAGACACT3′<br>R:5′ CAGATAGACAGCAAAAGCAAATA3′
OR10AD1	F:5′ GAGGCCCCCATAGTGATTGG3′<br>R:5′ AGAAAGTCTTCCCCCGACCT3′
OSCP1	F:5′ GCTCTACTCCAAGAAGGCCCT3′<br>R:5′ AAAGTGACCAGCAGCACATCC3′
FTL	F:5′ AGGCCTCCTACACCTACCTC3′<br>R:5′ ATGGCGTCTGGGGTTTTACC3′
CEBPB	F:5′ TTTGTCCAAACCAACCGCAC 3′<br>R:5′ GCATCAACTTCGAAACCGGC3′
TALDO1	F:5′ ACGCCATCGACGAGTACAAG 3′<br>R:5′ CTCGGCCCGGAATCTTCTTT3′
PPBP	F:5′ GGAAAGGAACCCATTGCA3′<br>R:5′ TCTGGGAGCATCTGGGTC3′
HCLS1	F:5′ ACAAGTCAGCAGTCGGCTTT 3′<br>R:5′ CGCTCTTATCCACTCGGTCC3′
TUBA1A	F:5′ AGAGGGTGAGGAAGAAGGAGA3′<br>R:5′ CAGAAATGGACAGCTTGGGT3′
CDKN2D	F:5′ CAACCGCTTCGGCAAGAC 3′<br>R:5′ TGTGACCCTCTTGAACTGC 3′
MSRB1	F:5′ GCTCGCTGAAGTTTGTCCCT3′<br>R:5′ CCTTGGGAGTGTCCTGATGT3′
RPS25	F:5′ CTGCGGTGTCTGCTGCTATT 3′<br>R:5′ CTTGTCCCGAACTTTGCCTT 3′
G0S2	F:5′ GTGCTCGGCCTGATGGAGACT 3′<br>R:5′ CTTCTGGAGAGCCTGTCGCTC 3′
